# Chronic Combined Oral Methylphenidate and Fluoxetine Increases Inflammation in Somatosensory and Mesolimbic Brain Regions

**DOI:** 10.1007/s11064-025-04608-3

**Published:** 2025-11-10

**Authors:** Julianna Roeser, Huy Lu, Abigail M. Lantry, Caleigh Hoerner, George Lagamjis, Shannon Klein, Rania Ahmed, Igor Elman, Albert Pinhasov, Ken Blum, Michael Hadjiargyrou, David E. Komatsu, Panayotis K. Thanos

**Affiliations:** 1 Behavioral Neuropharmacology and Neuroimaging Laboratory on Addictions, Clinical Research Institute on Addictions, Department of Pharmacology and Toxicology, Jacobs School of Medicine and Biomedical Sciences, University at Buffalo, Buffalo, NY 14068 USA; 2 Department of Psychology, City University of New York, Queens, NY USA; 3 Cambridge Health Alliance, Harvard Medical School, Cambridge, MA USA; 4 Department of Molecular Biology, Adelson School of Medicine, Ariel University, Ariel, Israel; 5 Division of Addiction Research & Education, Center for Sports, Exercise & Mental Health, Western University of the Health Sciences, Pomona, CA 91766 USA; 6 Department of Biological and Chemical Sciences, New York Institute of Technology, Old Westbury, NY USA; 7 Department of Orthopaedics and Rehabilitation, Stony Brook University, Stony Brook, NY USA; 8 Department of Exercise and Nutrition, University at Buffalo, 1021 Main Street, Buffalo, NY 14203-1016 USA

**Keywords:** Methylphenidate, Fluoxetine, Microglia, Neuroinflammation, Autoradiography, Serotonin reuptake inhibitors

## Abstract

Methylphenidate (MP) is commonly prescribed to treat attention deficit hyperactivity disorder (ADHD). ADHD and depression are often comorbid, leading to simultaneous use of serotonin reuptake inhibitors (SSRIs), such as Fluoxetine (FLX). Previous studies have shown MP increases microglial activation, which has been linked to neuroinflammation, but little is known about these two medications in combination. To address this gap in our knowledge, 3-week-old male Sprague Dawley rats were randomly assigned into four groups receiving either water, MP, FLX, or MP + FLX orally using a previously established dosing regimen. After four weeks of treatment the animal’s brains were collected for in vitro [^3^H] PK11195 autoradiography. Chronic treatment with MP and MP + FLX resulted in significantly increased [^3^H] PK11195 binding in somatosensory regions including the cortex limbs somatosensory (S(Limbs)), facial somatosensory (S(Face)), dorsal caudate putamen (D CPU), and ventral caudate putamen (V CPU). Chronic treatment with MP increased microglial activation in specific brain regions; however, these effects were not amplified by co-administration with fluoxetine. These findings emphasize the importance of further investigating the interactions between SSRIs and MP, particularly as their combined use becomes more prevalent.

## Introduction

Methylphenidate (MP) is a commonly prescribed psychostimulant used to treat attention deficit hyperactivity disorder (ADHD) in adolescents and adults. As of 2022, approximately 11.4% of children in the U.S. had received an ADHD diagnosis, with 77.9% having at least one co-occurring psychological disorder and 53.6% receiving ADHD medication [1]. ADHD diagnoses have been increasing over the past few decades and are expected to continue rising [2]. College students are also attracted to MP for recreational use due to its attention-focusing, weight loss, or euphoric effects, all of which raise concerns [3, 4]. MP functions by blocking dopamine (DA) and norepinephrine (NE) transporters in the brain thereby causing an increase in their extracellular concentrations [5].

Serotonin reuptake inhibitors (SSRIs) such as Fluoxetine (FLX) have been used to treat a variety of psychological conditions including major depressive disorder (MDD) in adolescents and adults [6]. Rates of adolescent depression are rising, with five million adolescents aged 12 to 17 experiencing at least one major depressive episode [7]. FLX is commonly prescribed in combination with MP in patients with both MDD and ADHD [8]. FLX blocks the serotonin (5-HT) reuptake transporter in presynaptic serotonin neurons, thereby increasing synaptic levels of 5-HT to relieve depressive symptoms [9].

The effects of MP and FLX are well understood when administered alone, but their combined use has not been thoroughly studied and requires further investigation. Previous studies have found that MP treatment increases DA transporter and dopamine type 1 receptor concentrations in the basal ganglia which may affect motor behavior while others found no effect [10, 11]. In adolescent rats, co-administration of MP and FLX has been shown to increase cocaine self-administration compared to MP or FLX alone, suggesting an increased addiction risk [12, 13]. Subgroups of rats receiving MP and FLX treatment also have been shown to have increased locomotion and stereotypies in open field tests, which could indicate functional deficits of basal ganglia circuits [13].

MP treatment has also decreased density in appendicular bones and reduced healing after injury [14, 15]. These effects were potentiated by the addition of FLX to the MP treatment [16]. It has been proposed that these effects are due to the reductions in body weight seen with treatment or may be due to dysregulation of osteoclast activity [16–18]. Combined MP and FLX treatment also produce differential changes in gene regulation in neuropeptides that act as direct and indirect pathway markers involved in sensorimotor and behavioral responses in the striatum [9, 19]. These changes in the striatum are associated with increased expression of the 5-HT1B receptor subtype [20]. Oral MP treatment also reduces N-methyl-D-aspartate (NMDA) receptor binding in rodent brains which could interfere with learning, memory and behavior [21]. Additionally, MP has been shown to affect the endocannabinoid system even after an abstinence period, which could alter physiological functioning and behavior [22].

Microglia are the immune cells in the central nervous system (CNS) and are important for immune responses and maintaining homeostasis by participating in the activation and regulation of neuroinflammation [23]. After sensing an injury, microglia undergo rapid activation and are believed to respond by increased phagocytosis and production of cytokines and chemokines, as well as potentially engaging in antigen presentation [24]. When overactivated, microglia release reactive oxygen species, nitric oxide and cytokines which may cause vascular damage or neurodegeneration [25]. PK 11,195 is a synthetic antagonist that selectively binds to translocator protein, also known as peripheral benzodiazepine receptor, which is upregulated in activated microglia following various neuronal injuries in rodent models [26–28]. Microglia-mediated neuroinflammation has also been associated with Alzheimer’s disease, Parkinson’s Disease, and amyotrophic lateral sclerosis (ALS) as well as other neurological diseases [25, 29].

Dopamine increases the levels of cytokines and chemokines in the brain, which cause an inflammatory response in the brain when dysregulated, activating microglial cells [30]. Increased extracellular dopamine is associated with MP use [5] and chronic daily treatment with MP for three months has been shown to increase microglial activation in multiple brain regions [31]. Conversely, FLX has been shown to have an anti-inflammatory effect under acute inflammatory stress [32] and was found to suppress microglia activation and inflammation in the postischemic brain [33]. FLX has also been shown to produce microglia-dependent neuroprotection against lipopolysaccharide induced neuronal damage [34]. Given these data, the current study aimed to assess the effects of combined oral MP and FLX treatment on neuroinflammation in rats using [^3^H] PK11195 autoradiography.

## Experimental Procedures

###  Animals

Adolescent three-week-old male Sprague Dawley rats were individually housed in humidity-controlled rooms (22 ± 2 ◦C, 50 ± 10% relative humidity) with 12-h reverse light-dark cycle (lights off at 0800 h). The animals had access to standard laboratory chow ad libitum. The rats were randomly assigned into four groups (*n* = 8/group) receiving water (control), MP, MP and FLX (MP + FLX) or FLX. Over a four-week period the animals received their respective treatments daily as previously established in a two-bottle eight-hour drinking paradigm. Animals were housed individually to ensure accuracy in fluid consumption and oral drug administration.

Rats in the MP group received 30 mg/kg of MP for one hour (9:00–10:00AM) and then received 60 mg/kg for seven hours (10:00AM-17:00PM). Rats in the MP + FLX group received 30 mg/kg of MP and 20 mg/kg FLX for one hour and then received 60 mg/kg of MP and 20 mg/kg FLX for the next seven hours. Rats in the FLX group received 20 mg/kg in two bottles, having one for one hour (9:00–10:00AM) and the second for seven hours (10:00AM-17:00PM) [35] [12, 36, 37]. The current study also utilized voluntary oral administration instead of intraperitoneal injection or gavage methods. These other methods could increase stress or offer less accurate results relating to humans [38]. At the end of treatment period, rats were euthanized with isoflurane (3.0%) and decapitation, their brains were isolated and flash frozen in 2-methylbutane and stored at −80 °C until utilized.

###  Drug Preparation

MP hydrochloride and FLX hydrochloride (Sigma Aldrich, St. Louis, MO) were dissolved in distilled water to produce stock solutions. The stock solutions were freshly diluted daily and final concentrations were prepared based on each animals’ body weight and average fluid consumption over the previous three days [12].

###  [^3^H] PK11195 Autoradiography

Brains were sectioned at 14 μm thick and mounted on slides. [³H] PK11195 binding was carried out as previously described [31, 39]. Sections were preincubated for 15 min in 50mM Tris-HCL buffer (pH 7.4) at room temperature. Sections were then incubated in 50mM Tris-HCL buffer (pH 7.4) with 0.8 nM [³H] PK 11,195 (85.7 Ci/mmol, PerkinElmer Inc.) for 30 min at room temperature. Nonspecific binding was determined on consecutive sections in the presence of an excess of 20µM unlabeled PK 11,195. After incubation, sections were washed twice for six minutes in ice-cold 50 mM Tris HCl buffer (pH 7.4) and then dipped in ice-cold distilled water.

Slides were then exposed to BioMax XAR film for four weeks alongside calibrated tritium standards (American Radiolabeled Chemicals, St. Louis, MO). The films were then developed and scanned as a TIFF image at 1200 DPI (Brother MFC-J651DW). Regions of interest include PRh (Perirhinal cortex), Ect (Ectorhinal cortex), PrL (Prelimbic cortex), Cg (Cingulate cortex), IL (infralimbic cortex), M (Motor cortex), Piri (Piriform cortex), Aud (Auditory cortex), Vis (Visual cortex), D CPU (dorsal caudate putamen), V CPU (ventral caudate putamen), NA (nucleus accumbens) GP (globus pallidus), Ent (entorhinal cortex), Rs (retrosplenial), S(Face) (facial somatosensory), S(limbs) (limbs somatosensory), S(Tr) (trunk somatosensory), S1/2 (somatosensory region), SNR (substania nigra), Amyg (amygdala), HP (hippocampus), Th (Thalamus), PAG (Periaqueductal gray), CB (cerebellum), and Colli (colliculi). ROIs were quantified using ImageJ software and expressed in µCi/g tissue.

###  Statistics

Specific [³H] PK11195 binding for each ROI was analyzed using one-way ANOVA for treatment effects followed by Tukey’s post hoc comparisons when needed. All statistical analyses and graphs were performed using GraphPad Prism 8 with statistical significance set at *α* = 0.05. Values are expressed as total [³H] PK11195 binding means (µCi/g) ± S.E.M.

## Results

**[³H] PK11195 Autoradiography**

**Fig. 1:**
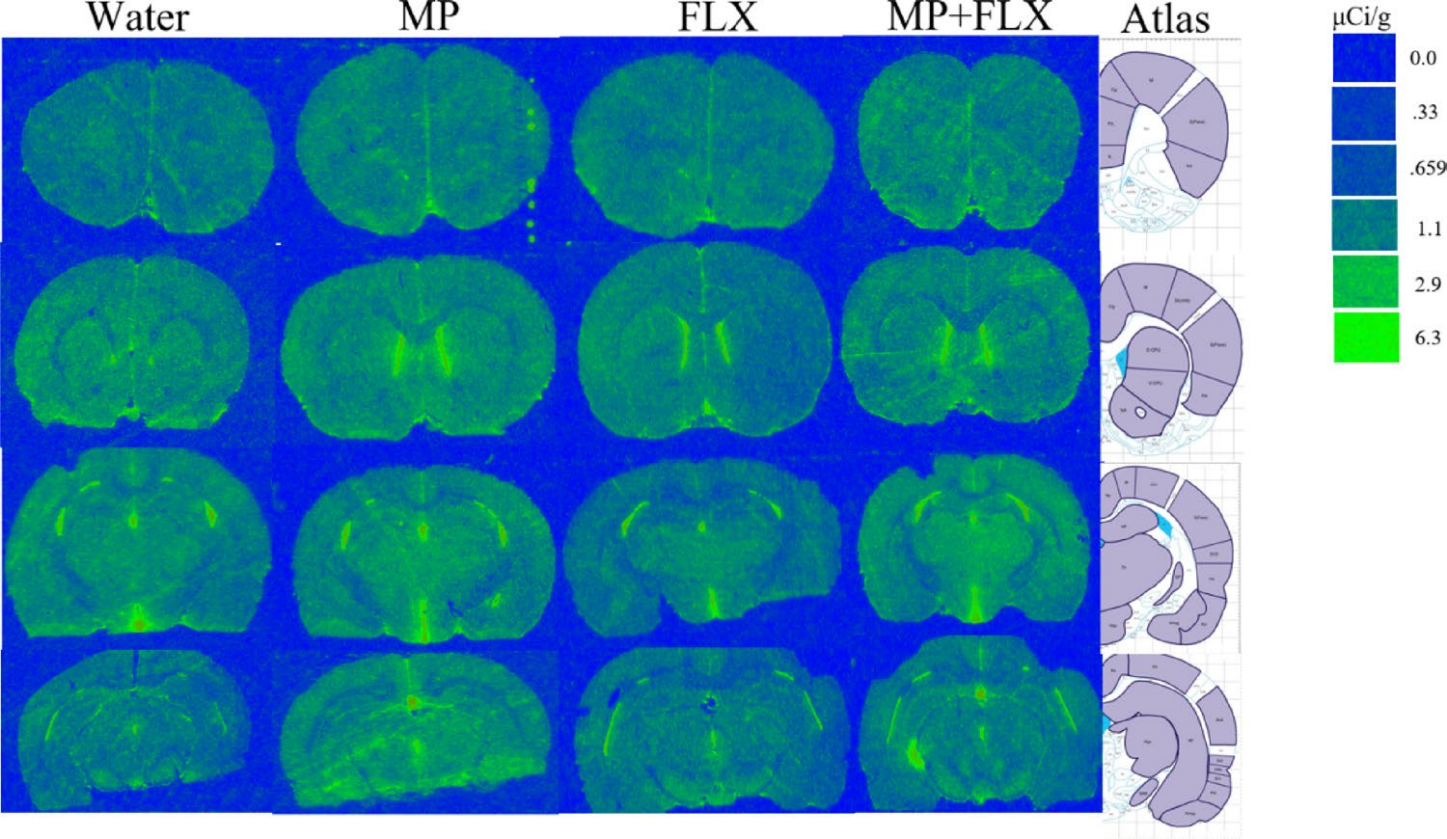
Representative autoradiographic coronal brain sections showing [³H] PK 11,195 binding following 4 weeks of drug treatment. Images corresponds to bregma coordinates: 3.00 m, 1.56 mm, −2.40 mm, −4.68 m with the corresponding regions of interest drawn onto the reference brain atlas images from Paxinos & Watson Rat Brain Atlas [40]

**Fig. 2:**
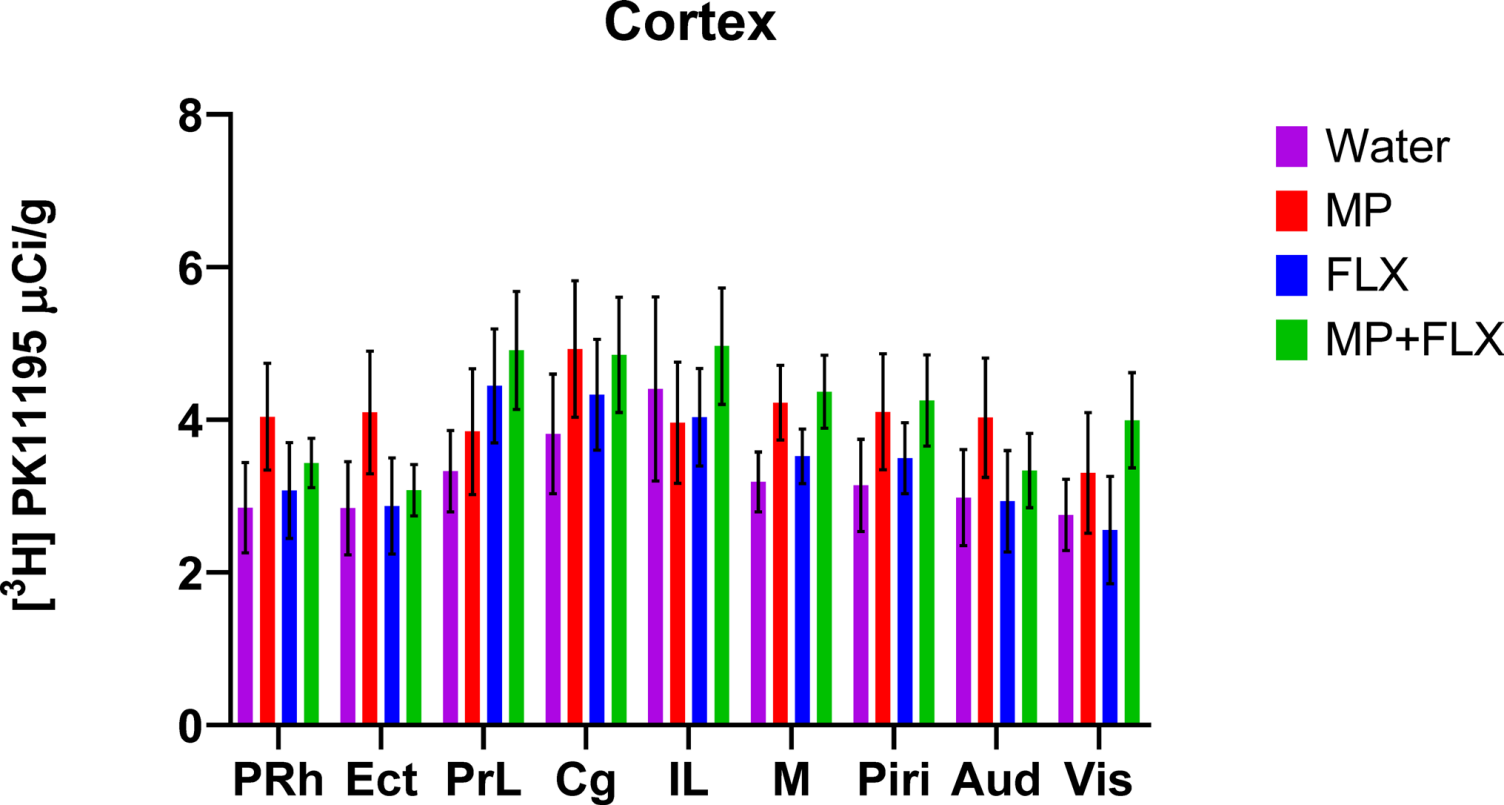
>Mean (± SEM) [³H] PK11195 specific binding levels in the cortex following four weeks of treatment in the PRh (Perirhinal cortex), Ect (Ectorhinal cortex), PrL (Prelimbic cortex), Cg (Cingulate cortex), IL (infralimbic cortex), M (Motor cortex), Piri (Piriform cortex), Aud (Auditory cortex), Vis (Visual cortex) across all treatment groups. No significant difference was observed (*p* > 0.05) across any of the groups with *n* = 8 per group

**Fig. 3:**
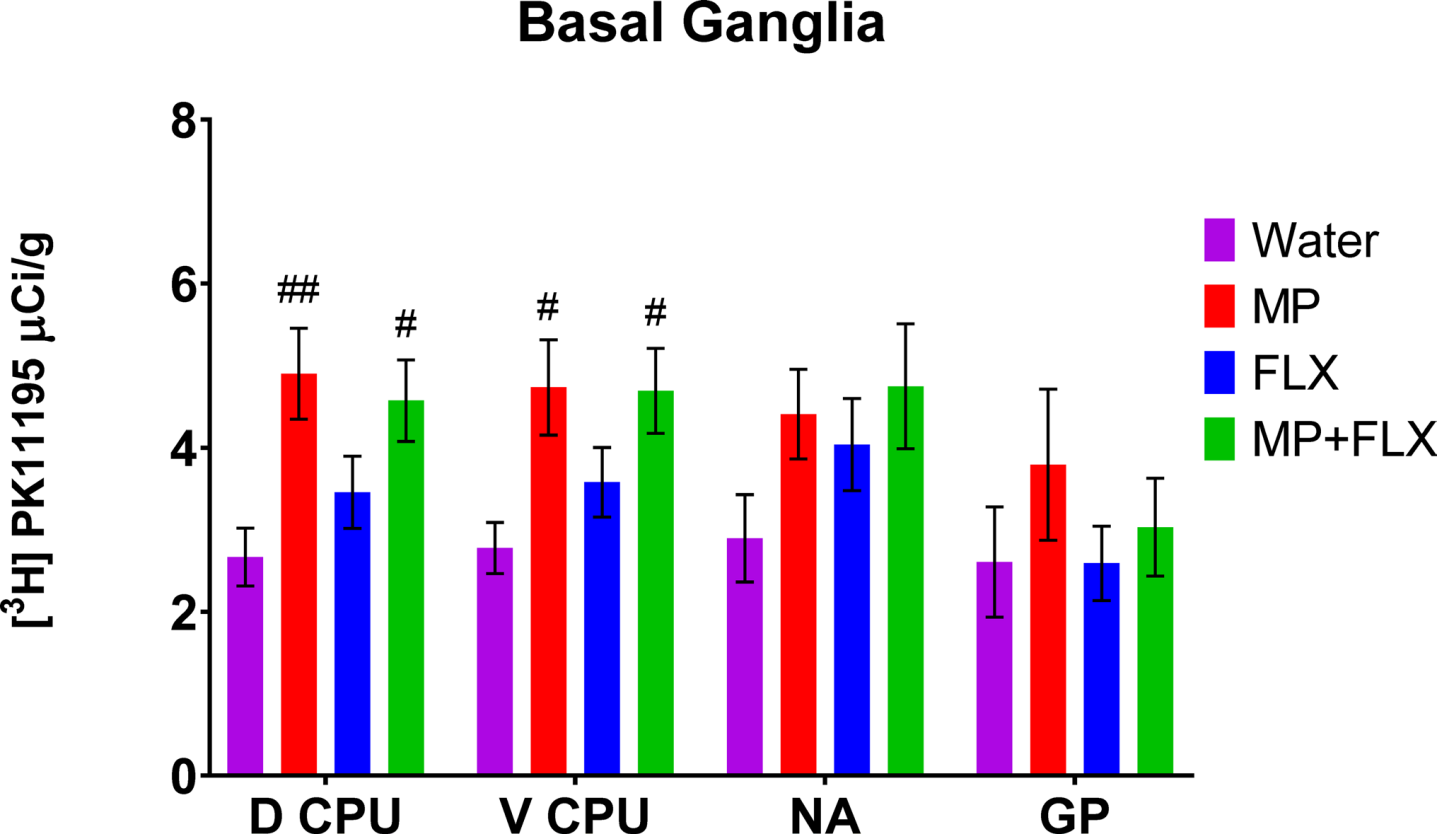
Mean (± SEM) [³H] PK11195 specific binding analysis of all treatment groups with *n* = 8 per group following 4 weeks of drug treatment of the basal ganglia within the D CPU (dorsal caudate putamen), V CPU (ventral caudate putamen), NA (nucleus accumbens) GP (globus pallidus). # denotes a significant difference compared to water (# *p* < 0.05, ## *p* < 0.01)

**Fig. 4:**
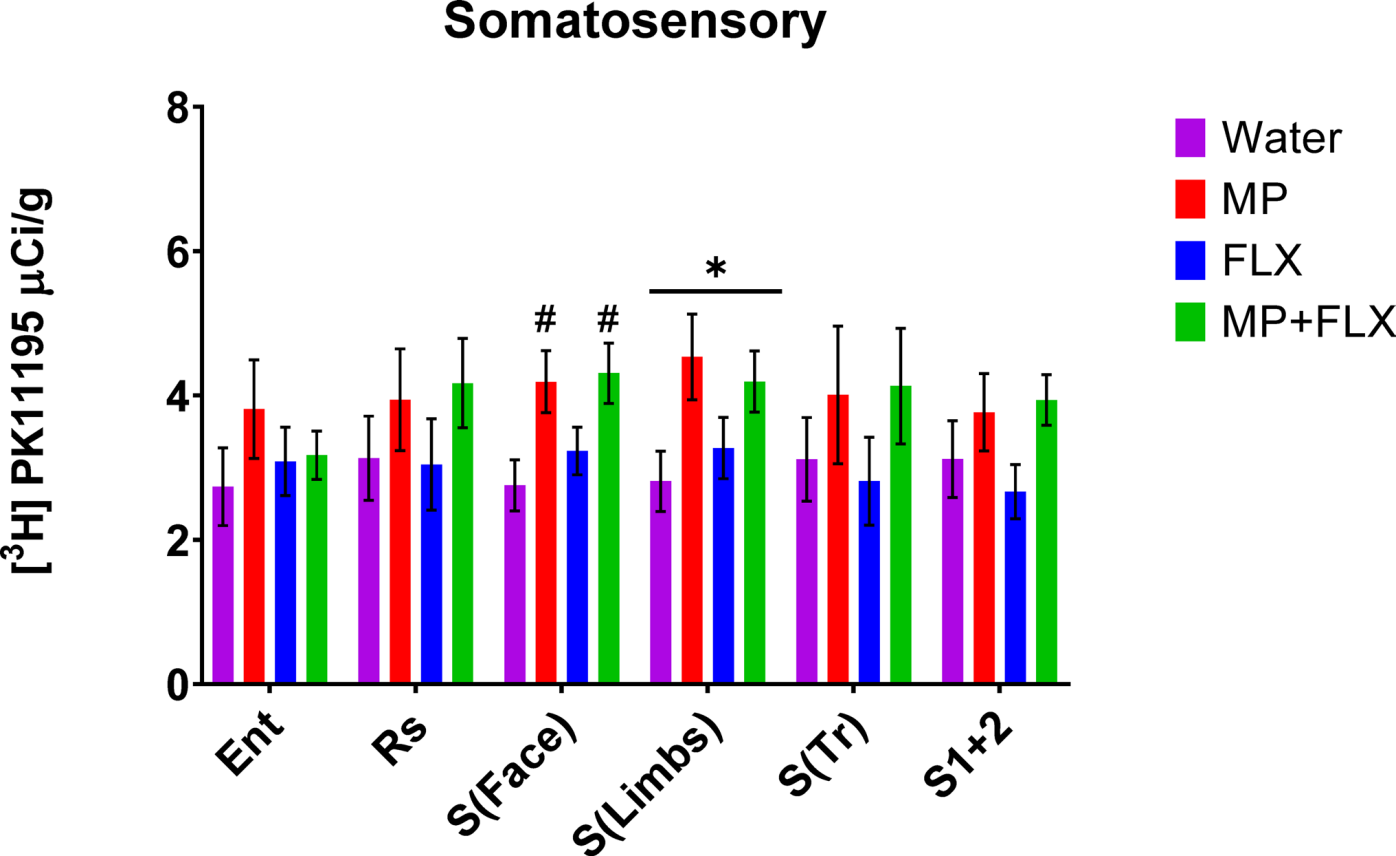
Mean (± SEM) [³H] PK11195 specific binding analysis of all treatment groups with *n* = 8 per group following four weeks of drug treatment of the somatosensory areas of the basal ganglia within the Ent (entorhinal cortex), Rs (retrosplenial), S(Face) (facial somatosensory), S(limbs) (limbs somatosensory), S(Tr) (trunk somatosensory), S1/2 (somatosensory region). * Denotes a significant difference (*p* < 0.05) in overall treatment effects. # denotes a significant difference between MP and MP + FLX compared to water

**Fig. 5:**
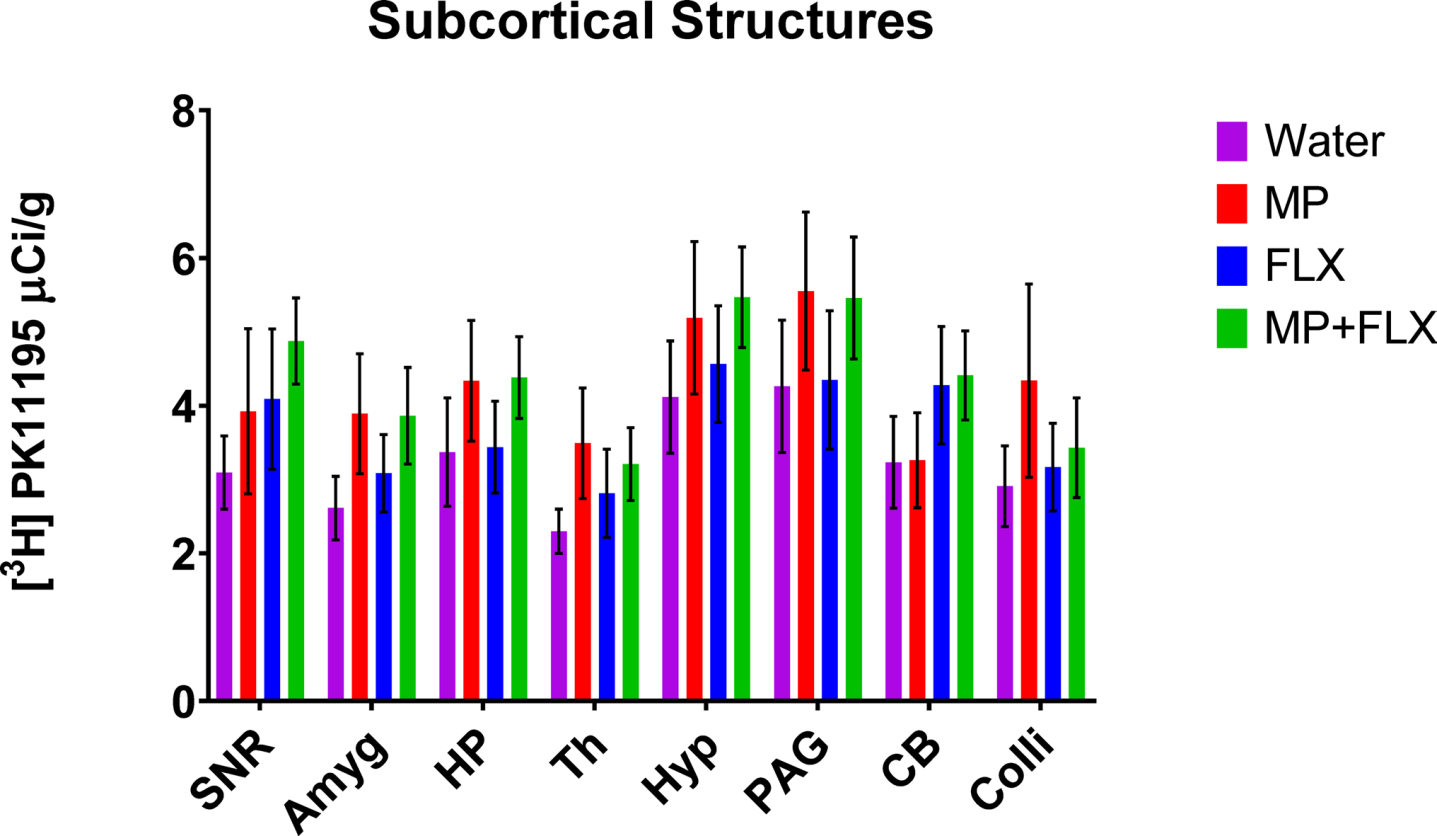
Mean (± SEM) [³H] PK11195 specific binding levels in the subcortical structures of the basal ganglia following four weeks of treatment in SNR (substania nigra), Amyg (amygdala), HP (hippocampus), Th (Thalamus), PAG (Periaqueductal gray), CB (cerebellum), and Colli (colliculi) across all treatment groups. No significant difference was observed (*p* > 0.05) across any of the groups with *n* = 8 per group. Each bar represents the group mean for [^3^H] PK11195 receptor binding

Specific binding was assessed for each ROI using [³H] PK11195 binding after four weeks of drug treatment (Fig. 1) and was analyzed with a one-way ANOVA with treatment groups (Water, MP, MP + FLX, and FLX) as a factor (Figs. 2–5). A significant effect of treatment was seen in the D CPU [F (3,90) = 4.657; *p* = 0.0045, Fig. 3], V CPU [F (3, 90) = 3.884; *p* = 0.0116, Fig. 3], S(Limbs) [F (3, 60) = 2.880; *p* = 0.0432, Fig. 4], and S(Face) [F (3,91) = 3.797; *p* = 0.0129, Fig. 4]. Tukey’s post hoc tests found a significant increase in binding in the MP group compared to the Water group in multiple regions including the D CPU (*p* = 0.0072, Fig. 3), V CPU (*p* = 0.0248, Fig. 3**)**, and S(Face) (*p* = 0.0490, Fig. 4). Tukey’s post hoc tests also found a significant increase in binding in the MP + FLX group compared to the Water group in regions including the D CPU (*p* = 0.0292, Fig. 3), V CPU (*p* = 0.0292, Fig. 3**)**, and S(Face) (*p* = 0.0279, Fig. 4).No significance was observed in all other ROIs (*p* > 0.05) (Figs 1, 2 and 5).

## Discussion

The current study investigated the effects of independent and combined dosing of MP and FLX on [³H] PK 11,195 binding in the brain. An increase in binding was seen in multiple regions throughout the brain in both the MP and MP + FLX groups including S(Face), S(Limbs), V CPU and D CPU after four weeks of drug treatment compared to the water control group. These results indicate chronic MP treatment can increase microglial activation and therefore neuroinflammation in certain brain regions but is not amplified by co-dosing with FLX. Also, FLX alone did not significantly affect microglial activation, either independently or in combination with MP. The MP + FLX group did not differ from the MP group in any region examined. This was unexpected as previous studies have shown FLX having a neuroprotective effect against inflammation and damage associated with microglial activation [33, 34]. This may be due to different administration routes, length of drug treatment or small sample size.

Microglia are immune cells that act as macrophages in the central nervous system when brain lesions or dysfunction occurs [41]. Microglia are also involved in healthy brain homeostasis, particularly in neuronal circuit formation and refinement by increasing neurogenesis and removing damaged cells as needed [42]. Additionally, they can promote cell proliferation and form neuronal circuits but can also cause central nervous system damage if activation is sustained [43]. When microglia become overactivated, they release reactive oxygen species, nitric oxide, and cytokines which are neurotoxic and cause damage to surrounding tissues [25]. Microglial activation has also been shown to occur after chronic MP [31] and methamphetamine use in rodents [39]. Methamphetamine increases extracellular dopamine levels [44], which may form toxic metabolites and induce neurotoxicity [45]. Like methamphetamine, MP has also been shown to increase free dopamine in the brain [5], potentially leading to increase in cytokine and chemokine levels, triggering an inflammatory response and activating microglia [30]. Microglia activation has been associated with Type 2 diabetes, Parkinson’s Disease, Alzheimer’s disease, Huntington’s Disease, Pick’s disease and ALS [25, 29].

Alzheimer’s disease is characterized by the accumulation of β-amyloid plaques and neurofibrillary tangles as well as microglial cell activation around these plaques [46]. Microglia are activated by β-amyloid and acute activation can aid in its clearance from the brain, slowing disease progression initially, offering a protective effect [47, 48]. Chronic microglia activation contributes to neurotoxicity and synapse loss through the release of proinflammatory cytokines, advancing the disease [48, 49]. Because of their involvement in Alzheimer’s disease pathogenesis, reducing or stopping the activation of microglia and the release of the inflammatory cytokines are being proposed as possible treatments [50, 51].

The caudate putamen (CPU) is involved in learning, motor control, reward, cognition, and addiction. Dysfunctions in this area have been linked to Parkinson disease, Huntington disease, Alzheimer’s disease, depression, Wilson disease, and autism [52]. In this study, increased [^3^H] PK11195 binding was found in both the ventral (V CPU) and dorsal (D CPU) in MP and MP + FLX groups compared to water control. The D CPU is involved in reward, memory, emotion, and decision-making functions [53]. Damage in this area has been linked to cognitive decline and executive dysfunction, especially in those with Parkinson’s disease [54]. MP alone has previously been shown to alter striatal dopamine type 2 receptor levels, impacting behavior and addiction risk [55]. When administered together, MP + FLX decreased dopamine type 2 receptors in the caudate putamen and nucleus accumbens more so than either drug administered alone, which could have implications in learning, memory, attention, sleep and reward seeking behavior [11]. The V CPU is involved in processing and regulating motor activity and expressing reward signals during motivated behaviors [56]. Lesions in ventral striatal regions have been associated with behavioral dysregulation, impulsivity, motivational deficits, and anxiety [54].

In the current study, we also detected increased binding in various sensorimotor areas including S(Face) and S(Limbs). Brain injury in somatosensory areas in rodents causes agitation when whiskers are stimulated compared to a soothing or indifferent response in uninjured animals [57]. In aging animals, difficulties walking and a reduction of sensory stimulation in the hindpaw have been related to plastic reorganization or degeneration [58]. In previous studies, norepinephrine levels were increased in somatosensory areas and long latency responses were repressed in rats treated with MP [59]. Brain glucose metabolism also is altered in sensory and motor areas after treatment with MP, with most effects subsiding after abstinence periods [60, 61].Children with ADHD have been shown to have deficiencies with somatosensory function and tactile perception, which have shown to be improved by treatment with MP [62, 63]. Further, MP enhances rodent performance in sensory-guided attention tasks by affecting sensory signal processing in the visual thalamus. Responses to the light stimulus demonstrated shorter latencies and were more robust when MP treatment was administered [64]. Rodent models also showed MP disrupted visual stimulus position discrimination tasks in Wistar-Kyoto rats more so than spontaneously hypertensive rats, indicating a neurochemical difference between the strains effecting treatment [65]. Previous studies showed that behavioral changes and neuroinflammation mediated by chronic oral MP administration can be reversed following prolonged abstinence, suggesting the current increase in [3 H] PK11195 binding observed in MP is reversible; however, the combined effect with FLX remains elusive and more research is needed [31, 66–68].

**Limitations**.

The current study focuses on the treatment effects of MP, FLX and their combination (MP + FLX) compared to a water control group. This study only utilized male rats and further studies with females are needed to determine potential sex differences in treatment response as previous studies have found sex differences in rodent behavior [69]. Rats in this study were single-housed in order to accurately measure fluid consumption and administer treatments which may influence results as this could be considered a deprived environment [70]. Future studies should also explore the effects of other SSRIs, such as vilazodone, co-administered with MP. Additionally, different treatment parameters, including drug doses treatment duration, film types and development times, should be considered in future studies.

**Future Directions**.

The number of ADHD diagnoses has been growing and is expected to rise further in coming years [2] along with the rates of non-prescription MP use in healthy individuals [71]. Adolescent depression is also on the rise [7], increasing the likelihood SSRIs and MP will be administered in combination. Non-prescription use of stimulants has also been associated with alcohol and marijuana dependence [72] further complicating neurological effects. Previous studies have also shown that chronic MP and FLX increase cocaine self-administration, indicating increased vulnerability to cocaine abuse [12]. It is becoming increasingly important to understand the combined effects of these medications at clinically relevant doses. Further research is needed not only to evaluate the effects of MP and FLX, but also to assess interactions with other SSRIs.

## Data Availability

Data is provided within the manuscript.
